# Peer Review of “State Anxiety Biomarker Discovery: Electrooculography and Electrodermal Activity in Stress Monitoring (Preprint)”

**DOI:** 10.2196/72093

**Published:** 2025-03-03

**Authors:** Daniela Saderi, Shailee Rasania, Toba Olatoye, Simon Muhindi Savai, Randa Salah Gomaa Mahmoud, Vasco Medeiros, Mitchell Collier

**Affiliations:** 1PREreview, Portland, OR, United States; 2Inflammatix, Inc, King of Prussia, PA, United States; 3Kwara State Teaching Service Commission, Ilorin, Nigeria; 4Zagazig University, Zagazig, Egypt

**Keywords:** stress, biomarker discovery, EOG, EEG, medical informatics, electrooculography, electroencephalography


*This is a peer-review report for the preprint “State Anxiety Biomarker Discovery: Electrooculography and Electrodermal Activity in Stress Monitoring.”*


This review is the result of a virtual collaborative live review discussion organized and hosted by PREreview and JMIR Publications on January 16, 2025. The discussion was joined by 16 people: 2 facilitators, 1 member of the JMIR Publications team, and 13 live review participants, including 3 who agreed to be named but have not contributed to composing this review into its final form: Uday Kumar Chalwadi, Killivalavan Solai, and Prasakthi Venkatesan. The authors of this review have dedicated additional asynchronous time over the course of 2 weeks to help compose this final report using the notes from the live review. We thank all participants who contributed to the discussion and made it possible for us to provide feedback on this preprint.

## Summary

Anxiety, particularly state anxiety (s-anxiety), is increasingly recognized as a health concern linked to mental and physical issues, including adverse cardiovascular and long-term health outcomes. This study [1] leverages noninvasive wearable technology to identify interpretable biomarkers resulting from s-anxiety using electrooculography (EOG) and electrodermal activity (EDA). Two datasets were developed: BLINKEO, focusing on blink-related EOG features, and EMOCOLD, analyzing EOG and EDA responses during a cold pressor test. The authors then used both datasets and applied statistical analysis (eg, *F*_1_-scoring, Shapley Additive Explanations [SHAP] analysis) to identify biomarkers of anxiety. Results revealed that using EOG data (blink duration, peak height, and opening integral) in tandem with EDA data (mean signal, permutation, entropy, and Hjorth activity) led to the identification of novel biomarkers that reveal nuanced emotional and stress responses. Moreover, it was found that SHAP analysis can more accurately determine which features are relevant to enhancing model performance. The findings highlight the potential of combining EOG and EDA biomarker data to create robust real-time models for anxiety detection. Combinations of physiological features (as sets) were more effective as measures of stress response than individual features alone. This research underscores the transformative role of noninvasive wearable technology in personalized mental health monitoring and intervention strategies.

## List of Major Concerns and Feedback

### Concerns With Methods

It would be helpful to document the name of the device and manufacturer used to record the EOG. This would help other researchers who may want to reproduce the results.Similarly, it would be helpful to add additional details about the cold pressor test methods. For example, was a commercially available circulating water bath used to maintain a constant water temperature? Was the temperature of the subject’s hand monitored? The details of the cold stressor test (the water temperature, the period of immersion, and the cutoff point) should be added for the sake of clarity, transparency, and reproducibility. Past studies using these metrics should also be referenced for details (eg, [2]). These methodological details may also be added in the form of a figure to add clarity to the experimental setup.To better understand the individual response to the cold challenge before participating in the actual experiment, it is advised that the manuscript states what type of participant testing was or was not adopted in the cold pressor testing experiment. For example, what were the tolerance times? Were there any gender differences? If any pretesting data were collected, analyzing them and presenting them as results would add clarity to the results.It is unclear if the 65 repeating blinking trials and the 19 no-blinking trials were collected from the same individual or from different individuals. Please clarify.No signal voltage/electrical records for EDA were found in the manuscript. Is this intentional? Please consider adding this information.It would be important to add details of ordinal variables present in the Positive and Negative Affect Schedule and the State-Trait Anxiety Inventory (STAI-State), and clearly state their function and use in Supplementary Table 2.

### Concerns With Analysis

*F*_1_-scores that were mentioned in the text (87.34% and 79.99%) are not present within the figures. Moreover, an *F*_1_-score is an integer value from 0 to 1, taking precision and recall into account, and is not often expressed as a percentage.Figure 1c has two separate graphs; it should be captioned as 1c and 1d. What do both these graphs portray? The second graph for 1c is missing titles for the x- and y-axes—the current assumption is that they are the same as the first graph.Table 1 lacks a legend and is shown as panel a of Table 2. Please check how the tables are referenced in the text to make sure they reference the right one.The captions of the figures should have statistical information when relevant. For example, in Figure 3, the caption should include a description of what data were plotted and the meaning of the graph. Presumably plotting medians, quartiles, and SDs? Also, please report n values.

### Concerns With Ethics

It is not clear what the ethical statement at the end of the manuscript, which states that the study was exempt from review board approval, means. That statement should be revised for clarification. In addition, details regarding whether or not institutional review board approval was obtained, whether the study involved consenting participants and used humans, how the data were collected and used, how the data were handled to protect the privacy of study participants, and any other ethical procedures that were followed to protect subjects from any harm due to participation in the study should be added.

## List of Minor Concerns and Feedback

### Minor Concerns With Methods

Please document whether the data were taken from each subject only once or whether data were obtained several times from a subject.Referring to the line “To focus on blink-like events, we applied criteria based on established blink characteristics,” the criteria used to establish blink characteristics should be cited, if not already given.SHAP analysis was performed on combinations of 5 features. Please clarify on what basis these 5 features were chosen (out of 15 of EDG and 33 of EOG).

### Minor Concerns With Analysis and Presentation

Page 10, Electrooculography (EOG) Signal Segmentation section: the authors mentioned that they extracted 33 features; however, Supplementary 4 mentioned 35 feature definitions. Please revise and correct.In Figure 3, please put “STAI-State survey score” on the y-axis for clarification rather than just “Scores.” In addition to box and whiskers plots, adding column graphs for positive affectivity, negative affectivity, and s-anxiety might be beneficial to more clearly express the SD present within the data.It would be beneficial to graphically display the *F*_1_-scores that were collected across the study.The figures are quite small, which makes readability a little difficult. Please make the text larger to improve readability and accessibility.The Figure 1a description states, “The red dotted lines indicate the center of the peak…,” but these appear to be gray.

### Suggestions

Consider the inclusion of a Limitations section in this manuscript to better discuss potential limitations due to the skewness in male and female participants, data curation, applied methodologies, and other limitations of the study.A figure showing the trial structure would be very useful to understand how the data were collected.

## References

In the third paragraph of the Introduction, adding a reference to other techniques used to provoke anxiety, including the reduced EDA response in depressed patients, and the conflicting studies could be helpful to the readers.In the Introduction, fourth paragraph, the reference “Schachter and Singer” is not present in the References. Is this the wrong reference, or it just needs to be added to the list?In the Introduction, third page, third paragraph, it is advised to add references to document the reduced EDA response in depressed patients and the conflicting studies.In the Methods, please cite sources for the Butterworth filter (page 5), the Savitzky-Golay filter (page 5), and all other analyses.Reference 2: Include full citation with a link.Reference 3: It is advised to correct the article name to “APA 2023 Stress in America Topline Data.”Reference 4: The correct citation should be “Kazanskiy NL., Khonina S.N., Butt M.A. A review on flexible wearables—Recent developments in non-invasive continuous health monitoring. Sens. Actuators A Phys. 2024;366:114993. doi: 10.1016/j.sna.2023.114993.”Reference 10: The correct citation should be: “Electrooculogram Analysis and Development of a System for Defining Stages of Drowsiness Master's Thesis Project in Biomedical Engineering, Linköping University, Dept. Biomedical Engineering, LiU-IMT-EX-351 Linköping 2003. Available: https://www.diva.portal.org/smash/get/diva2:673960/FULLTEXT01.pdfTest”Reference 19: The correct citation should be “Anxiety Detection Using Multimodal Physiological Sensing, 2021 IEEE EMBS International Conference on Biomedical and Health Informatics (BHI), Athens, Greece, 2021, pp. 1-4, doi: 10.1109/BHI50953.2021.9508589.”Reference 23: Revising this citation is advised as searching on the internet shows error 404. The requested URL was not found on this server. Moreover, this is not a proper citation—give the edition number of the book (there are at least 5 editions) and publication year, as well as the page number of the cited data point about typical blink elapsed time.Reference 27: The correct citation should be “Hassanein, A.M.D.E., Mohamed, A.G.M.A. & Abdullah, M.A.H.M. Classifying blinking and winking EOG signals using statistical analysis and LSTM algorithm. Journal of Electrical Systems and Inf Technol 10, 44 (2023). https://doi.org/10.1186/s43067-023-00112-2.”In general, citations need to be reviewed and added with consistency throughout the manuscript.
